# Nasopharyngeal Carcinoma: Clinical Achievements and Considerations Among Treatment Options

**DOI:** 10.3389/fonc.2021.635737

**Published:** 2021-11-29

**Authors:** Zheran Liu, Ye Chen, Yonglin Su, Xiaolin Hu, Xingchen Peng

**Affiliations:** ^1^ Department of Biotherapy, Cancer Center, West China Hospital, Sichuan University, Chengdu, China; ^2^ Department of Medical Oncology, Cancer Center, the State Key Laboratory of Biotherapy, West China Hospital, West China Medical School, Sichuan University, Chengdu, China; ^3^ Department of Rehabilitation, Cancer Center, West China Hospital, Sichuan University, Chengdu, China; ^4^ Department of Nursing, West China Hospital, Sichuan University, Chengdu, China

**Keywords:** nasopharyngeal carcinoma, intensity-modulated radiotherapy, chemotherapy, targeted therapy, immunotherapy

## Abstract

Nasopharyngeal carcinoma (NPC) is a severe malignancy arising from the nasopharyngeal epithelium and is southern China’s third most common cancer. With the advancement of treatment methods, early-stage NPC patients usually have a better prognosis and more prolonged survival period than those with other malignant tumors. Most treatment failures are due to distant metastasis or a locally advanced stage of NPC in the initial diagnosis. In addition, approximately 10% of patients develop local recurrence, and 10%–20% of patients experience distant metastasis after treatment. These patients have a poor prognosis, with a median survival of only approximately 10–15 months. In the rapid development of treatment options, the efficacy and safety of some treatments have been validated and approved for first-line treatment, while those of other treatments remain unclear. The present study aims to provide a comprehensive overview of recent advances in NPC treatment and explain the various therapeutic possibilities in treating locally advanced, recurrent, and metastatic NPC patients.

## Introduction

Nasopharyngeal carcinoma (NPC) is a malignant tumor arising from the nasopharyngeal epithelium. Globally, there were 129,079 new cases of NPC and 72,987 deaths in 2018 (1). NPC has a unique geographical and ethnic distribution. The incidence of NPC is less than 1/100,000 in Caucasians, while the prevalence of NPC is much higher in East and Southeast Asians. NPC is the third most common cancer in southern China, with an incidence of 2–10/100,000 (2, 3).

At present, radiotherapy (RT) is the only curative treatment for NPC. Intensity-modulated radiotherapy (IMRT) has become one of the most important achievements in the treatment of NPC. NPC usually infiltrates and grows near critical tissues and organs, including the brain stem, spinal cord, optic nerves, and optic chiasm (4). Compared with traditional two-dimensional radiotherapy (2D-RT), IMRT can deliver tumoricidal doses to the tumor while reducing the doses received by the adjacent normal tissue (5).

However, the 5-year survival rate is only 67%–77% when patients receive RT alone for locoregionally advanced NPC (6, 7). RT combined with chemotherapy, including concurrent, adjuvant, and induction chemotherapy, has been introduced as a standard treatment. RT combined with chemotherapy can increase survival rates and decrease distant metastatic rates for locoregionally advanced NPC, while the optimal combination of treatments still needs to be defined. Furthermore, distant metastases and locoregional recurrence are still two dominant failure patterns for NPC patients (8, 9). Targeted therapy and immunotherapy have shown promising clinical efficacy in recurrent or metastatic NPC. The present article reviews the recent progress in the management of NPC and aims to clarify the direction of clinical research.

## IMRT

Compared to traditional 2D-RT, IMRT can bring clinical benefits with less treatment-associated toxicity in NPC patients. A prospective, randomized clinical trial (RCT) recruited 616 nonmetastatic stage I to IVb (according to the American Joint Committee on Cancer [AJCC] 6th edition staging system) NPC patients to investigate the difference in the clinical outcomes and toxicities between 2D-RT and IMRT (10). The results illustrated that the IMRT group had much better 5-year local control [hazard ratio, (HR) = 1.67, 95% CI = 1–2.77] and overall survival (OS, HR = 1.77, 95% CI = 1.25–2.51) rates than the 2D-RT group. In the IMRT group, the rates of acute complications (including xerostomia and oral mucositis) and late complications (including temporal lobe neuropathy, cranial nerve palsy, trismus, neck fibrosis, late xerostomia, and hearing loss) were markedly decreased (10). Zhang et al. retrospectively analyzed 7,081 nonmetastatic NPC patients, including 2,245 IMRT-treated patients and 4,838 2D-RT-treated patients. They showed that the 5-year local relapse-free survival, locoregional relapse-free survival, progression-free survival (PFS), and OS rates of IMRT-administered patients were significantly better than those of 2D-RT-administered patients (11). IMRT is also recommended for child and adolescent NPC patients. Qiu et al. demonstrated that IMRT-treated children and adolescents had a significantly higher OS, distant metastasis-free survival, and locoregional relapse-free survival than 2D-RT-treated children and adolescents. For adverse effects, no more acute toxicities occurred, while for late complications, the rate of grade 2–4 xerostomia and hearing loss were reduced in the IMRT group (12). Generally, the clinical benefits of IMRT have overwhelming superiority compared with 2D-RT. Based on the supporting evidence, for all NPC patients, IMRT with daily image guidance should be implemented according to CSCO (The Chinese Society of Clinical Oncology) and ASCO (American Society of Clinical Oncology) guidelines (13).

Can IMRT bring better local control and survival benefits than 3D-CRT? Zhang et al. conducted a meta-analysis that included eight previous studies and compared the survival outcomes of 948 IMRT-treated patients with 1,176 2DRT/3D-CRT-treated patients. Treating NPC patients with IMRT was associated with a better 5-year OS (HR = 1.51, 95% CI = 1.23–1.87) and tumor local control (odds ratio [OR] = 1.94, 95% CI = 1.53–2.46) than treating patients with 2DRT/3D-CRT. Moreover, IMRT showed great benefits in reducing radiation-induced chronic toxicities such as trismus (OR = 0.18, 95% CI = 0.04–0.83) and temporal lobe neuropathy (OR = 0.44, 95% CI = 0.28–0.69) (14). However, this meta-analysis mixed 2D-RT and 3D-CRT into the same control group, making the comparison between IMRT and 3D-CRT alone obscure. Fang et al. suggested that there was no significant difference between the 3D-CRT- and IMRT-treated groups in the 3-year locoregional control, metastasis-free survival, and OS rates (84.8% vs. 84.2%, 76.7% vs. 82.6%, and 81.7% vs. 85.4%, respectively, all *p* > 0.05), but the IMRT-treated group had a better quality of life than the 3D-CRT-treated group, especially in the acute toxicity recovery stage (15). Kuang et al. suggested that compared to 3D-CRT, IMRT provided a better 4-year locoregional tumor control rate (93.6% vs. 85.3, *p* = 0.012) and OS (83.5% vs. 72.1%, *p* = 0.036) with fewer side effects (16). Multi-institutional retrospective research conducted by Sung Ho Moon et al. analyzed 1,237 NPC patients and revealed that IMRT had a significantly better 5-year OS than 3D-CRT (70.7% vs. 57.8%, *p* = 0.011) (17). Thus, generally, using IMRT has more benefits than using 3D-CRT.

## Proton Therapy

Although IMRT delivers therapeutic doses to tumors near critical structures in the skull, the irradiation of adjacent tissues is unavoidable because of the inherent restriction arising from the physical properties of the photon beam (18). On the other hand, proton beam therapy has shown better conformality because the properties of the Bragg peak allow maximum doses to be deposited on the target cancer. The toxicity of proton beam therapy is also less than that of IMRT because proton beam therapy can eliminate the exit dose beyond the target area (19). In 2021, a propensity score-matched study included 80 NPC patients who received intensity-modulated proton therapy (IMPT) and 278 patients who were treated with volumetric modulated arc therapy (VMAT) (20). IMPT was associated with a lower incidence of nasogastric tube placement and body weight loss because of the lower mean oral cavity dose (20). Li et al. compared the toxicity and survival outcomes of 77 patients with nonmetastatic NPC treated with IMPT or IMRT. The results showed that IMPT was associated with lower grade 2–4 acute adverse events and better oncological outcomes than IMRT, including 100% locoregional control at 2 years (21). Given the dosimetric advantages and reduced damage to normal tissue, proton therapy may be potentially suitable for patients with recurrences who received prior radiotherapy because reirradiation with photon-based therapy might result in a high risk of complications and morbidities (22). Verma et al. systematically assessed the clinical outcomes and adverse events of patients who received proton radiotherapy for reirradiation (23). They concluded that irradiation using proton-based RT for head and neck cancer exhibited a good locoregional control rate and less toxicity (23). In summary, proton therapy has revealed broad therapeutic prospects and might be suitable for reirradiating patients with locoregional recurrences. More information from both prospective and retrospective studies is still warranted to provide more substantial evidence to support the role of proton therapy in the treatment of NPC patients (19).

## Concurrent Chemoradiation

IMRT has been recommended as the standard treatment for stage II–IV NPC patients (24). However, IMRT alone cannot significantly reduce the distant metastasis rate, which is still the major cause of patient death (25). Distant metastasis is also the major failure pattern of NPC, especially in locoregionally advanced NPC patients (26–28). Concurrent chemoradiation therapy (CCRT) is suitable when treating locoregionally advanced NPC patients (24). The phase III Intergroup-0099 laid the status of CCRT (29). According to this trial, the 3-year PFS rate of RT alone was only 24%, while for CCRT, the rate was 78%, and similar results were also shown in the 3-year overall survival rate (46% vs. 76%, respectively) (29). Ribassin-Majed et al. conducted an individual patient data network meta-analysis to compare all available treatments for locally advanced NPC (30). In general, their results showed that CCRT has favorable survival outcomes compared with radiotherapy alone (30). Other RCTs further showed the advantages of CCRT over RT alone in considering PFS and overall survival (31–33). In 2018, He et al. reviewed the existing evidence and performed a comprehensive meta-analysis based on 15 randomized controlled trials comprising 1,142 patients with locoregionally advanced NPC to compare the efficiency and toxicity of using RT alone or CCRT. The results revealed that CCRT could improve the overall response rate (risk ratio, RR = 0.53, 95% CI = 0.43–0.66) and the complete response rate (RR = 0.60, 95% CI = 0.51–0.71) (34). However, the incidence of adverse events (except grade 3–4 gastrointestinal reaction) increased, including the incidence of hematological toxicity (*p* < 0.0001), radiation-induced oral mucositis (*p* = 0.007), and radiodermatitis (*p* = 0.01) (34). In 2021, the CSCO and ASCO guidelines recommended CCRT for all NPC patients without contraindications (13).

Since the administration of cisplatin-based CCRT may increase the adverse events of NPC patients, will new-generation antitumor platinum-based drugs reduce the adverse effects and maintain satisfactory efficacy? Nedaplatin is a second-generation platinum-based chemotherapy drug and an analog of cisplatin. Nedaplatin has similar effects and is associated with fewer digestive symptoms and renal toxicity than cisplatin in the clinic. A phase II clinical trial confirmed that the regimen of induction chemotherapy with docetaxel plus nedaplatin followed by concurrent nedaplatin and IMRT is as effective as cisplatin-based induction chemotherapy and CCRT with even better tolerance and patient compliance (35). Furthermore, a phase III trial including 402 stage II–IVB (according to the AJCC 7th edition staging system) NPC patients compared the efficacy and safety of nedaplatin-based concurrent chemoradiotherapy with those of cisplatin-based concurrent chemoradiotherapy (36). The trial findings suggested that PFS and OS were comparable between these two treatment regimens, and nedaplatin-based concurrent chemoradiotherapy was associated with a lower frequency of grade 3 or 4 late auditory or hearing toxicities (36).

## Target Drugs Replaced Chemotherapeutic Drugs in CCRT

EGFR-based CCRT is another emerging alternative concurrent chemotherapy regimen that may be appropriate treatment for advanced-stage NPC patients. The discussion of the comparison of treatment outcomes and toxicities of EGFR-based CCRT with cisplatin is still ongoing. At present, there are two main types of clinical drugs that can inhibit EGFR. One is monoclonal antibodies (e.g., cetuximab and nimotuzumab), and the other is small-molecule tyrosine kinase inhibitors (e.g., gefitinib). As an IgG1 monoclonal antibody, cetuximab was the first clinically verified EGFR inhibitor.

The efficacy and safety of additional anti-EGFR antibodies to standard treatment and additional concurrent anti-EGFR antibodies to radiotherapy have been evaluated by Peng et al. by literature-based meta-analyses (37). The results showed that an anti-EGFR antibody could significantly improve OS (HR = 0.51, 95% CI = 0.39–0.66) and DFS (HR = 0.68, 95% CI = 0.54–0.86) compared to RT or chemoradiotherapy alone, but concurrent anti-EGFR antibody plus radiotherapy failed to achieve survival benefits compared with traditional cytotoxic-drug-based CCRT in locoregionally advanced NPC patients (37). For the assessment of the antitumor efficacy of the additional anti-EGFR antibody to cytotoxic regime-based CCRT, a phase II study demonstrated the clinical efficiency of treating locoregionally advanced NPC patients with concurrent cetuximab, cisplatin, and IMRT in stage III, IVa, and IVb (according to the AJCC 7^th^ edition staging system) NPC patients. The results demonstrated that concurrent cetuximab and cisplatin with IMRT had 86.5% 2-year disease-free survival (DFS) in treating locoregionally advanced NPC with accepted side effects (38). Eighty-seven percent of patients showed grade 3–4 oropharyngeal mucositis, and 10% of patients had grade 3 cetuximab-related acneiform rash (38). Recently, You et al. recruited stage II–IVb (according to the AJCC 7th edition staging system) NPC patients to compare the treatment efficacy between the CCRT alone group and the CCRT plus cetuximab group. The results showed that the CCRT plus cetuximab group had better OS, DFS, and distant metastasis-free survival (39). However, in the same year, Li et al. performed a case–control study to compare the efficacy and toxicities of applying cisplatin-based CCRT alone and concomitant cetuximab for NPC patients in stage II to IVb (according to the AJCC 7th edition staging system). The result was the opposite of the conclusion of You et al. It suggested that CCRT plus cetuximab had no significant benefit in improving the OS, PFS, and distant metastasis-free survival of NPC patients (CCRT plus cetuximab vs. CCRT alone: 89.7% vs. 90.7%, 83.9% vs. 88.7%, and 88.4% vs. 91.9%, respectively). In addition, CCRT plus cetuximab may even aggravate acute mucositis and acneiform rash (40). In 2018, Wang et al. conducted a meta-analysis including 783 qualified patients. The results showed that both the complete response rate (RR = 1.97, 95% CI = 1.57–2.46) and the response rate (RR = 1.32, 95% CI = 1.20–1.46) of patients treated with CCRT plus cetuximab were significantly better than those of patients treated with CCRT alone, while the 3-year OS rate (RR = 1.09, 95% CI = 0.86–1.39) and side effects were not different between the two groups (41). The main toxicities were mucositis (RR = 1.27, 95% CI = 0.86–1.88) and rash (RR =1.46; 95% CI = 0.88–2.44). However, this study had a strong selection bias because all the patients were from China. Thus, the pros and cons of using cetuximab with CCRT still need to be further discussed.

In addition, VEGF-targeted drugs may also boost the efficacy of CCRT. Bevacizumab is a restructured humanized monoclonal VEGF-targeted antibody with positive therapeutic outcomes in multiple cancers, including colorectal cancer, lung cancer, breast cancer, and renal cancer (42–45). The application of adding bevacizumab to cisplatin and radiotherapy for stage III–IV disease could enhance the treatment effects with manageable toxicities (46). Another phase II RCT evaluated the clinical effects of adding bevacizumab to cisplatin-based CCRT. The 2-year locoregional progression-free survival, distant metastasis-free survival, PFS, and OS rates were 83.7%, 90.8%, 74.7%, and 90.9%, respectively, and no unusual grade 3–4 toxicity events were observed (47). Thus, the results showed that bevacizumab had strong associations with a better treatment outcome when added to CCRT.

## Additional Adjuvant Chemotherapy After CCRT for Locoregionally Advanced NPC

The role of adjuvant chemotherapy in treating locoregionally advanced NPC is ambiguous. Although the Intergroup-0099 study demonstrated an improved 3-year OS rate when applying CCRT followed by adjuvant chemotherapy, the control group used RT alone. Thus, whether the survival benefits were acquired from concurrent chemotherapy or adjuvant chemotherapy is still unclear. The efficacy of adjuvant chemotherapy is questioned in the IMRT era. Subsequent studies failed to confirm the positive influence of adjuvant chemotherapy (48–50). Chen et al. found that adjuvant cisplatin 80 mg/m^2^ and fluorouracil treatment 800 mg/m^2^/day failed to achieve better survival by conducting a multicenter RCT. They reported no significant difference in the 5-year failure-free survival rate (75% vs. 71%), OS (83% vs. 80%), DFS (85% vs. 80%), or locoregional failure-free survival (91% vs. 90%) between additional adjuvant chemotherapy with cisplatin and fluorouracil and CCRT alone (51). In addition, a high incidence of grade 3–4 peripheral neuropathy was observed in the CCRT followed by the adjuvant chemotherapy group (2% vs. 0.4%) (51). Furthermore, a meta-analysis that pooled 8,036 NPC patients also demonstrated that adjuvant chemotherapy had no survival benefits in OS, PFS, distant metastasis-free survival, and locoregional recurrence-free survival (52).

Moreover, Chan et al. investigated the potential effects of adjuvant chemotherapy on plasma Epstein–Barr virus (EBV)-detectable NPC patients. Patients with screened post-RT EBV DNA received an additional six cycles of cisplatin and gemcitabine. The results showed no relapse-free survival benefits in NPC patients with detectable post-IMRT seropositive EBV RNA compared with undetectable EBV DNA patients (HR = 1.09, 95% CI = 0.63–1.89) (53). The dose reduction because of the patients’ intolerance to adjuvant chemotherapy after CCRT and the failure to eliminate micrometastases using a cisplatin and fluorouracil combination might be the leading causes of failure in applying adjuvant chemotherapy.

Above all, selecting the most suitable patients and identifying the most effective drug combination for adjuvant chemotherapy are the top problems. For the first problem, omics characteristics such as miRNA or radiomics signatures might be helpful to determine the most suitable patients for adjuvant chemotherapy (54). For the second problem, the efficacy of the development of chemotherapy drugs such as capecitabine and tegafur, or biotherapy drugs such as PD-1/L1, and CTL4 inhibitors need further evaluation. Findings from a recent phase III RCT suggested that additional metronomic capecitabine as an adjuvant therapy could significantly improve failure-free survival in patients with locoregionally advanced NPC compared with standard therapy (CCRT with or without induction chemotherapy, HR = 0.5, 95% CI = 0.32–0.79) (55). These results supported that adjuvant therapy using metronomic capecitabine might have a promising future in the treatment of locoregionally advanced NPC (55).

## Additional Induction Chemotherapy Followed by CCRT for Locoregionally Advanced NPC

Induction chemotherapy followed by sequential CCRT has shown potential benefits for patient survival, which was confirmed by a series of RCTs (**Table 1**). Chen et al. pooled individual patient data from four randomized trials in endemic areas involving 1,193 NPC patients to evaluate the efficiency of applying induction chemotherapy to CCRT. The data revealed that induction chemotherapy plus CCRT was superior to CCRT alone with improved PFS (HR = 0.70, 95% CI = 0.56–0.86) and OS (HR = 0.75, 95% CI = 0.57–0.99) (65). Updated meta-analyses also confirmed these results (66, 67).

**Table 1:** Recently randomized controlled trials comparing the induction chemotherapy plus concurrent chemoradiotherapy with concurrent chemoradiotherapy alone in locally advanced nasopharyngeal carcinoma.

Study	Phase	Sample size	Induction chemotherapy regimen at experimental arm	Concurrent chemotherapy regimen at control group	Median PFS (95% CI)	PFS HR (95%CI)	Median OS (95% CI)	OS HR (95% CI)
Hui et al. (56)	Phase II	65	Docetaxel 75 mg/m^2^ on day 1 and cisplatin 75 mg/m^2^ on day 1 every 3 weeks for 2 cycles	Cisplatin 40 mg/m^2^ every week for 8 weeks	3-year: 88% *vs*. 60%	0.49 (0.20–1.19)	3-year: 94% *vs*. 68%	0.24 (0.08–0.73)
Fountzilas et al. (57)	Phase II	141	Cisplatin 75 mg/m^2^ on day 2, epirubicin 75 mg/m^2^ on day 1, and paclitaxel 175 mg/m^2^ on day 1 every 3 weeks for 4 cycles	Cisplatin 40 mg/m^2^ every week	3-year: 65% *vs*. 64%	1.40 (0.71–2.77)	3-year: 67% *vs*. 72%	0.95 (0.48–1.89)
Tan et al. (58)	Phase II/III	172	Gemcitabine 1,000 mg/m², carboplatin AUC = 2.5, paclitaxel 70 mg/m² on day 1 and day 8 every 3 weeks for 3 cycles	Cisplatin 40 mg/m^2^ every week for 8 weeks	3-year DFS: 75% *vs*. 67%	0.77 (0.44–1.35)	3-year: 94% *vs*. 92%	1.05 (0–2.19)
Li et al. (59)	Phase III	480	Docetaxel 60 mg/m^2^ on day 1, cisplatin 60 mg/m^2^ on day 1, and fluorouracil 600 mg/m^2^/day on days 1–5 every 3 weeks for 3 cycles	Cisplatin 100 mg/m^2^ every 3 weeks for 3 cycles	5-year FFS: 77% *vs*. 66%	0.67 (0.48–0.91)	5-year: 86% *vs*.77%	0.65 (0.43–0.98)
Yang et al. (60)	Phase III	476	Cisplatin 80 mg/m^2^ on day 1, fluorouracil 800 mg/m^2^/day on days 1–5 every 3 weeks for 2 cycles	Cisplatin 80 mg/m^2^ every 3 weeks for 3 cycles	5-year DFS: 73% *vs*. 63%	0.66 (0.48–0.89)	5-year: 81% *vs*. 77%	0.69 (0.49–0.98)
Frikha et al. (61)	Phase III	83	Docetaxel 75 mg/m^2^ on day 1, cisplatin 75 mg/m^2^ day 1, and fluorouracil 750 mg/m2/day on days 1–5 every 3 weeks for 3 cycles	Cisplatin 40 mg/m^2^ every week	3-year: 74% *vs*. 57%	0.44 (0.20–0.97)	3-year: 86% *vs*. 69%	0.40 (0.15–1.04)
Hong et al. (62)	Phase III	479	Mitomycin 8 mg/m^2^, epirubicin 60 mg/m^2^, and cisplatin 60 mg/m^2^ on day 1, fluorouracil 450 mg/m^2^, and leucovorin 30 mg/m^2^ on day 8 every 3 weeks for 3 cycles	Cisplatin 30 mg/m^2^ every week	5-year DFS: 61% *vs*. 50%	0.74 (0.57–0.97)	5-year: 72% *vs*. 68%	0.92 (0.67–1.27)
Zhang et al. (63)	Phase III	480	Gemcitabine 1,000 mg/m^2^ on days 1 and 8, cisplatin 80 mg/m^2^ on day 1 every 3 weeks for 3 cycles	Cisplatin 100 mg/m^2^ every week for 3 cycles	3-year RFS: 85% *vs*. 77%	0.51 (0.34–0.77)	3-year: 95% *vs*. 90%	0.43 (0.24–0.77)
Lee et al. (64)	Phase III	802	Cisplatin 100 mg/m^2^, either fluorouracil 1,000 mg/m^2^/day for 120 h or capecitabine 2,000 mg/m^2^/day for 14 days every 3 weeks for 3 cycles	Cisplatin 100 mg/m^2^ every 21 days for 2 to 3 cycles	5-year: 75% *vs*. 69% (control group: concurrent-adjuvant sequence)	0.84 (0.65–1.08)	5-year: 82% *vs*.77% (control group: concurrent-adjuvant sequence)	0.82 (0.62–1.08)

PFS, progression-free survival; OS, overall survival; HR, hazard ratio.

PF (cisplatin plus 5-FU), TP (docetaxel plus cisplatin), and TPF (docetaxel, cisplatin, and fluorouracil) are available for the current regimen options for induction chemotherapy. In 2016, a phase III, multicenter, randomized controlled trial performed by Sun et al. recruited 480 stage III–IVB (except T3-4N0) untreated NPC patients and reported that a three-cycle TPF regimen followed by CCRT was an effective regimen for locoregionally advanced NPC. The results showed that this regimen significantly improved the 3-year failure-free survival (80% vs. 72%), OS (92% vs. 86%), and distant failure-free survival (90% vs. 83%) compared with CCRT alone (68). In 2018, the same induction regimen was confirmed to improve the PFS of patients (HR = 0.44, 95% CI = 0.20–0.97) (61). Yang et al. compared the survival outcomes of PF-based CCRT with CCRT alone in locoregionally advanced NPC. The results showed that PF-based CCRT provided significantly prolonged long-term OS, DFS, and distant metastasis-free survival with no difference in grade 3–4 adverse events compared with CCRT alone (69). The survival benefits were confirmed by a meta-analysis conducted by Maïmouna et al., which showed that the addition of induction therapy to CCRT could significantly improve OS (HR = 0.68, 95% CI = 0.511–0.91) and PFS (HR = 0.66, 95% CI = 0.57–0.76) (70).

Currently, cisplatin-based induction chemotherapy plus CCRT has been recommended by the National Comprehensive Cancer Network (NCCN) clinical practice guidelines for locoregionally advanced NPC (71). Lobaplatin, a third-generation platinum drug, might be an alternative to cisplatin-based induction therapy. Lv et al. conducted a phase III study that evaluated the treatment efficacy between lobaplatin-based and cisplatin-based induction therapy in the treatment of locoregional advanced NPC. The results showed that lobaplatin-based induction therapy could achieve similar survival (5-year PFS: HR = 0.98, 95% CI = 0.69–1.39) and fewer toxic effects than cisplatin-based therapy (72).

However, which regimen has the maximum efficacy for NPC patients is still controversial. A head-to-head study comparing the outcomes is still needed for comparison. He et al. performed a network meta-analysis involving seven RCTs with 1,570 patients (73). TPF was the most effective regimen for improving the OS of locoregionally advanced NPC patients (HR = 0.68, 95% CI = 0.42–1.1). However, the adverse effects of TPF were also severe. A significant increase in hematological toxicity incidence was observed. Moreover, several phase II RCTs showed that applying gemcitabine plus cisplatin as induction chemotherapy could enhance patient survival (74, 75). Zhang et al. performed a multicenter phase III RCT to compare the survival outcomes between gemcitabine plus cisplatin as induction chemotherapy followed by CCRT versus CCRT alone. The results showed that recurrence-free survival and overall survival in the gemcitabine plus cisplatin group were significantly improved (3-year recurrence-free survival: HR = 0.51, 95% CI = 0.34–0.77; 3-year overall survival: HR = 0.43, 95% CI = 0.24–0.77) (63).

## Targeted Therapy and Immunotherapy for Recurrent and Metastatic NPC

Up to 6% of NPC patients had distant metastasis at the initial diagnosis. These patients have a poor prognosis with a median survival of approximately 10–15 months (76). In addition, approximately 10% of patients will develop local recurrence after treatment, and 10%–20% of patients will develop distant metastasis (77–79). However, recommended treatments for recurrent and metastatic NPC patients are still lacking. Generally, the emerging treatment options for recurrent and metastatic NPC patients include chemotherapy using gemcitabine plus cisplatin, targeted therapy using EGFR or VEGFR, and immunotherapy such as immune checkpoint inhibitors (ICIs).

Zhang et al. recruited 362 recurrent or metastatic NPC patients and randomly assigned them to the gemcitabine plus cisplatin treatment group and fluorouracil plus cisplatin treatment group in a 1:1 ratio. The median PFS time in the gemcitabine plus cisplatin group was significantly prolonged compared with that in the fluorouracil plus cisplatin group (7.6 months vs. 5.6 months, HR = 0.55, 95% CI = 0.44–0.68). The OS of HR = 0.55, 95% CI = 0.44–0.68 was also improved (HR = 0.62, 95% CI = 0.45–0.84). Although the adverse events were similar between the two groups, those in the gemcitabine plus cisplatin group were mainly related to hematological toxicity, while the adverse events in the fluorouracil plus cisplatin group were mainly related to mucosal inflammation (80). Recently, Ma et al. evaluated the outcomes of several standard first-line chemotherapies for recurrent or metastatic NPC patients. The treatments include PF, GP, taxanes plus platinum, and triplet combination regimens (81). The results showed that the taxanes plus platinum regimen had the best long-term efficacy, followed by GP (81). In 2020, You et al. evaluated the efficacy and safety of conducting locoregional IMRT after PF chemotherapy in patients with chemotherapy-sensitive metastatic NPC. The results showed that chemotherapy followed by locoregional IMRT could significantly improve OS (HR = 0.42, 95% CI = 0.23–0.77) and PFS (HR = 0.36, 95% CI = 0.23–0.57) (82). Recently, VEGF-targeted therapies have been clinically tested for NPC patients. VEGF, a promoter of angiogenesis, participates in the formation of blood vessels. Studies have proven that VEGF is overexpressed in 2/3 of NPC patients, and VEGF overexpression is related to lymph node metastasis, NPC recurrence, and poor prognosis (83). Clinical trials confirmed that upregulated VEGF was related to worse survival time, anti-VEGF treatment was efficient for inhibiting tumor angiogenesis and promoting tumor cell apoptosis, and anti-VEGF treatment could even reverse resistance to chemotherapy for patients with locoregionally advanced NPC (83, 84).

Sunitinib is an oral small-molecule multitarget receptor tyrosine kinase (RTK) inhibitor that can inhibit VEGFR1–3. Sunitinib was approved for treating advanced renal cell carcinoma and imatinib mesylate-resistant or intolerant gastrointestinal stromal tumors (85). Hui et al. followed 13 patients with recurrent or metastatic NPC who had previously received high-dose radiation and found modest clinical activity with a high incidence of high-grade hemorrhagic events after applying sunitinib. Patients with local tumors invading the carotid sheath should be given special attention because of the risk of fatal hemorrhage (86). Another angiogenesis inhibitor, pazopanib, reported similar results. Lim et al. performed a phase II study in which they collected 33 recurrent or metastatic NPC patients with a daily dose of 800 mg of pazopanib (87). No complete response was observed. The median OS was 10.8 months (95% CI = 8.6–21.8 months), with 44% 1-year OS and 13% PFS (87). Generally, pazopanib showed encouraging activity in NPC patients with an acceptable toxicity profile.

In general, VEGF-targeted therapies exhibit certain treatment responses with better survival outcomes in treating recurrent or metastatic NPC patients. However, the increased risk of hemorrhage may lead to severe consequences and thus needs to be given additional attention. The current findings still require validation, and further investigation is warranted.

ICIs are currently an emerging strategy to treat recurrent or metastatic NPC patients. Several RCTs have been conducted to examine the efficacy and safety of ICIs for recurrent or metastatic NPC patients (**Table 2**). The antitumor activity and adverse effects of pembrolizumab, a humanized PD-1 antibody, were assessed by the KEYNOTE-028 study (88). In the KEYNOTE-028 study, 27 PD-L1-positive unresectable or metastatic NPC patients who failed prior treatments were recruited, and the results showed that the median PFS and OS for patients receiving pembrolizumab were 6.5 and 16.5 months, respectively. Grade 3–4 drug-related adverse events occurred in eight (29.6%) patients. Pembrolizumab might be a potential option for recurrent or metastatic NPC with acceptable toxicity (88). Another phase II RCT investigated the clinical outcome of nivolumab in treating recurrent or metastatic NPC patients. The 1-year OS rate was 59% (95% CI = 44.3%–78.5%), and the 1-year PFS rate was 19.3% (95% CI = 10.1%–37.2%) (89). In 2019, Fang et al. reported the results of two single-arm phase I trials that evaluated the antitumor efficacy and adverse effects of camrelizumab. In one trial, previously treated recurrent and metastatic NPC patients were enrolled, and in another trial, treatment-naïve recurrent and metastatic NPC patients in China were included. The results of the two RCTs demonstrated that both camrelizumab monotherapy and camrelizumab with gemcitabine and cisplatin had tolerable toxicity and potential antitumor activity in treating recurrent or metastatic NPC patients (90). Since adding camrelizumab to gemcitabine and cisplatin has shown good antitumor activity, a recent phase III RCT named CAPTAIN-1st compared camrelizumab versus placebo in combination with gemcitabine and cisplatin as first-line treatment for recurrent or metastatic NPC patients. The results showed that the camrelizumab group had significantly longer PFS than the placebo group (HR = 0.54, 95% CI = 0.39–0.76), showing that camrelizumab plus gemcitabine and cisplatin could become a promising standard treatment for recurrent or metastatic NPC patients (92).

**Table 2:** Completed clinical trials evaluating PD-1 inhibitors in recurrent and/or metastatic nasopharyngeal carcinoma.

Study	Trial identifier	Key eligibility criteria	Sample size	Dose	ORR	Median PFS (95% CI)	Median OS (95% CI)	Grade 3 or higher AE
Hsu et al. (88)	NCT02054806	Unresectable or metastatic disease; failure on standard therapy before; PD-L1 expression in 1% or more of tumor cells or tumor-infiltrating lymphocytes	27	Pembrolizumab 10 mg/kg every 2 weeks up to 2 years	26%	16.5 (10.1 to NR)	6.5 (3.6 to 13.4)	15/27
Ma et al. (89)	NCT02339558	Recurrent and/or metastatic disease; received at least one prior line of platinum-based chemotherapy	44	Nivolumab 3 mg/kg every 2 weeks on a 4-week cycle	21%	2.8 (1.8 to 7.4)	17.1 (10.9 to NR)	10/44
Delord et al. (89)	NCT02488759	Recurrent and/or metastatic disease; received less than two prior-line of systemic therapies	24	Nivolumab 240 mg every 2 weeks	21%	2.4 (1.5 to NR)	NR	2/24
Fang et al. (90)	NCT02721589	Recurrent and/or metastatic disease; failure at platinum-based chemotherapy	93	Camrelizumab at the prespecified doses of 1 mg/kg, 3 mg/kg, and 10 mg/kg, and a bridging dose of 200 mg per dose once every 2 weeks	34%	5.6 (3.3 to 7.9)	NR	15/93
Fang et al. (90)	NCT03121716	Recurrent and/or metastatic disease; previously untreated	23	Camrelizumab 200 mg on day 1, gemcitabine 1 g/m² on days 1 and 8, and cisplatin 80 mg/m² on day 1 every 3 weeks for six cycles, followed by camrelizumab 200 mg maintenance once every 3 weeks	91%	NR	NR	20/23
Wang et al. (91)	NCT02915432	Recurrent and/or metastatic disease; failure at prior standard chemotherapy, or disease progression within 6 months after adjuvant chemotherapy or chemoradiotherapy	190	Toripalimabis 3 mg/kg once every 2 weeks	21%	1.9 (1.8 to 3.5)	17.4 (11.7 to 22.9)	27/190
Yang et al. (92)	NCT03707509	Recurrent and/or metastatic disease; previously untreated	134	Camrelizumab 200 mg every 3 weeks, cisplatin 80 mg/m^2^ on day 1 of each 21 day, at most 6 cycles, gemcitabine 1,000 mg/m^2^, day 1 and day 8 of each 21 days, maximum 6 cycles	87%	9.7 (8.3 to 11.4)	NR	126/134
Even et al. (93)	NCT02605967	Nonkeratinizing recurrent and/or metastatic NPC, failure at platinum-based chemotherapy	82	Spartalizumab 400 mg once every 4 weeks	18%	1.9 (1.8 to 3.5)	25.2 (NR to NR)	37/87

NR, not reached; PFS, progression-free survival; OS, overall survival; AE, adverse event; ORR, overall response rate.

Several clinical trials have focused on using immunotherapy together with CCRT or induction chemotherapy. Moreover, combined therapy using nivolumab and ipilimumab, a CTLA-4 inhibitor, is ongoing. Generally, checkpoint inhibitors show promising antitumor effects in treating recurrent and metastatic NPC. However, the use of these checkpoint inhibitors in the real world still lacks evidence.

## Conclusion

IMRT is widely used for NPC patients, especially in the early stage. For locoregionally advanced NPC patients, the combination of chemotherapy and IMRT is recommended. CCRT could significantly improve the survival outcome of patients. Targeted drugs or second-generation platinum-based chemotherapy drugs can reduce the adverse effects compared with platinum-based CCRT. Until now, the treatment benefits of adjuvant chemotherapy have seemed obscure. The ways to screen NPC patients suitable for adjuvant chemotherapy and identify the efficacy of new chemotherapy drugs, including EGFR inhibitors or PD-1 antibodies, need further exploration. For induction chemotherapy, head-to-head comparisons of the treatment outcomes of different regimens are still lacking. Additionally, the optimal number of chemotherapy cycles needs further confirmation. Targeted therapy and immunotherapy might be the last treatment option for metastatic or recurrent NPC patients. To date, targeted therapy and immunotherapy have shown preliminary antitumor effects, and the adverse effects were acceptable. More evidence is needed to guide the next steps in the clinical application of immunotherapy.

## Author Contributions

All authors contributed equally in the writing of the review article. All authors contributed to the article and approved the submitted version.

## Funding

The work was supported by the National Natural Science Foundation of China (82172842, 81803104, and 81672386), the Sichuan Province Science and Technology Support Program (2021YFSY008 and 2020YFS0276), the West China Nursing Discipline Development Special Fund Project (HXHL21008), the Technology Innovation Project of Chengdu Science and Technology Bureau (2019-YF05-00459-SN), and the Postdoctoral Research and Development Fund and Translational Medicine Fund of West China Hospital (2020HXBH119 and CGZH19002). The funders had no role in study design, data collection and analysis, decision to publish, or preparation of the manuscript.

## Conflict of Interest

The authors declare that the research was conducted in the absence of any commercial or financial relationships that could be construed as a potential conflict of interest.

## Publisher’s Note

All claims expressed in this article are solely those of the authors and do not necessarily represent those of their affiliated organizations, or those of the publisher, the editors and the reviewers. Any product that may be evaluated in this article, or claim that may be made by its manufacturer, is not guaranteed or endorsed by the publisher.
